# Combined effects of maternal supplementation of iron, calcium, folic acid, and multivitamin during pregnancy on obesity in Chinese preschoolers born macrosomia

**DOI:** 10.3389/fped.2025.1608521

**Published:** 2025-06-27

**Authors:** Ming-Fei Yan, Esben Strodl, Wei-Kang Yang, Xiao-Na Yin, Guo-Min Wen, Deng-Li Sun, Dan-Xia Xian, Ya-Fen Zhao, Wei-Qing Chen

**Affiliations:** ^1^ Department of Epidemiology, School of Public Health, Sun Yat-Sen University, Guangzhou, China; ^2^ School of Psychology and Counselling, Queensland University of Technology, Brisbane, QLD, Australia; ^3^ Women’s and Children’s Hospital of Longhua District of Shenzhen, Shenzhen, China; ^4^ School of Health, Xinhua College of Guangzhou, Guangzhou, China

**Keywords:** obesity, supplementation during pregnancy, iron, calcium, folic acid, multivitamin, preschoolers, macrosomia

## Abstract

**Objective:**

Childhood obesity has become a global public health crisis. Previous studies have shown that nutritional supplementation during pregnancy may be protective against offspring obesity. However, the research in this area is still emerging and the impact of moderators, such as birth weight, upon outcomes has not been fully explored. This study aimed to examine the combined effect of maternal supplementation with iron, calcium, folic acid, and multivitamin during pregnancy on the risk of obesity in Chinese preschoolers born macrosomia.

**Methods:**

A total of 6,031 singleton children, born macrosomia, aged 3–6.5 years old were recruited from Longhua District in Shenzhen of China in 2021. Their mothers were asked to complete a structured questionnaire for collecting the sociodemographic characteristics of the child and parents, the child's birth-related characteristics, and maternal supplementation with iron, calcium, folic acid, and multivitamins during pregnancy. The children's weight and height were measured using a standardized method by well-trained medical staff from the Women's and Children's Hospital of Longhua District of Shenzhen.

**Results:**

After controlling for confounding variables, including other nutrients, the results of a series of logistic regressions showed that only iron supplementation (AOR = 0.75, 95% CI = 0.60–0.92) during pregnancy was negatively associated with the presence of obesity in preschoolers born macrosomia in boys. In contrast, there was no independent associations between maternal prenatal ingestion of iron, calcium, folic acid, or multivitamin supplements and obesity in preschool girls born macrosomia. Examination of interaction effects through crossover analyses showed that maternal supplementation with both iron and calcium (AOR = 0.68, 95% CI = 0.49–0.94), and both iron and multivitamins (AOR = 0.64, 95% CI = 0.48–0.86) during pregnancy significantly reduced the risk of obesity in male preschoolers born macrosomia. Furthermore, interaction analysis found a multiplicative interaction between maternal iron and multivitamin supplementation during pregnancy on the risk of obesity in male preschoolers born macrosomia (IOR = 0.52, 95% CI = 0.35–0.79).

**Conclusion:**

Our findings suggest that iron supplementation during pregnancy may reduce the risk of obesity in preschoolers born macrosomia in boys, with this effect enhanced with the combined ingestion of calcium and multivitamin supplementation.

## 1 Introduction

The worldwide public health crisis of childhood obesity has become a reality. Overweight and obese children had an increase in prevalence from 4.2% in 1990 to 6.7% in 2010, as revealed by cross-sectional studies in 144 countries, which represents a relative increase of 60% (1). In 2016, the number of obese girls and boys worldwide exceeded 50 million and 74 million respectively (2). By 2030, it is expected that the prevalence rate of obesity in Chinese children aged 0–7 will hit 6.0%, meaning that the prevalence of obesity among Chinese children will rise to 6.64 million (3). Wang et al. conducted a survey in Chinese aged 3–7 years and found that there was a 10.38% obesity prevalence rate among preschoolers, with the detection rate of obesity in boys was twice as high as that in girls (4). Overweight or obese children are more prone to psychiatric disorders such as depression, anxiety, and low self-esteem (5, 6), and more susceptible to a variety of metabolic and cardiovascular diseases including hypertension, hyperlipidemia, and type 2 diabetes mellitus (7–10). Moreover, a cohort study found that about three-fourths of overweight or obese children continued to have excess weight into adulthood (11). As such, obesity in childhood results in significant lifetime economic and social costs, including increased spending on health care, reduced wages, and reduced likelihood of employment (12–15). It is therefore critical to identify risk and protective factors for childhood obesity to steer the creation of potent public health measures.

Childhood obesity is the result of a multifactorial combination of genetics, prenatal and postnatal lifestyle, and the environment. In terms of genetic factors, the genes most studied in relation to obesity include the *FTO* and *MC*4*R* genes, while the *TCF7l, MTNR1B, ADRB3, INSIG2, APOB48R* genes have been related to glucose and lipid metabolism (16). Prenatal factors like mother's pre-pregnancy BMI and father's BMI have been strongly associated with offspring obesity (17). The risk of obesity increases for children born via cesarean section compared to those born via vaginal delivery (18, 19). In addition, rapid maternal weight gain during pregnancy, environmental tobacco exposure and maternal alcohol consumption affect fetal metabolism and growth and further result in increased rates of childhood obesity (20–23). Similarly, excessive catch-up growth of premature infants and low birth weight infants can lead to childhood obesity (24). Furthermore, an earlier meta-analysis demonstrated that macrosomia was an independent risk factor for obesity (25). In addition, postnatal influences on childhood obesity have been well studied. For example, the short duration of breastfeeding, irrational feeding practices, short sleep, low physical activity and long screen time exacerbated the likelihood of obesity in children (26–28).

Macrosomia, a term used to describe a fetus that weighs more than or equal to 4 kg at birth, is associated with gestational diabetes mellitus, pre-pregnancy weight, weight gain during pregnancy, and nutritional factors during pregnancy (29). In recent years, the prevalence of macrosomia has been 8.7% in China (30). A similar incidence rate of 8.07% has recently been found in the United States (31). These high incidence rates are concerning given that macrosomia increases the incidence of cesarean section, perinatal and neonatal complications, compared with normal birth weight fetuses (32, 33). Macrosomia have been exposed to an adverse environment of hyperinsulinemia and hyperglycemia in the uterus, resulting in increased fat and protein storage in the fetus (34). According to the Development Origins of Health and Disease Hypothesis (DOHaD) (35), malnutrition during fetal development can promote obesity through metabolic, genetic and behavioral changes. Studies based in France (36) and China (37) have found that higher birth weight and macrosomia are positively correlated with high BMI growth trajectory patterns of children in the early postnatal period, a prospective cohort study in Asia have suggested that the rapid growth pattern of BMI trajectory in infancy and early childhood will increase the risk of overweight/obesity in children in the future (38). Macrosomia is known to be an independent risk factor for childhood obesity, but not all children born macrosomia will become obese. Early nutrition may influence the incidence of macrosomia and subsequent metabolic patterns through persistent changes in DNA methylation, increasing susceptibility to certain chronic diseases (39, 40). Given that early nutrition can have an impact on the formation of macrosomia and may also have an effect on childhood obesity after birth, it is very necessary to pay attention to the future obesity situation of this special group of macrosomia. So the authors wondered what role nutritional factors play in childhood obesity in macrosomia.

Maternal malnutrition during pregnancy can lead to adverse birth outcomes such as macrosomia and low birth weight infants, and an increased risk of childhood obesity (41–43). Iron is a component of hemoglobin. The decrease in hemoglobin concentration due to increased blood volume during pregnancy leads to maternal iron-deficiency anemia. Prior studies have shown that the correlation between pregnant women's hemoglobin concentration and adverse birth outcomes in offspring is U-shaped (41, 42). The risk of future metabolic diseases, including obesity, could be reduced by taking iron supplementation during pregnancy (44). Calcium supplementation during pregnancy might also be beneficial in reducing the risk of gestational diabetes (GDM) and hypertensive disorders of pregnancy (45, 46). Maternal calcium dysfunction directly affects the synthesis and metabolism of fat in offspring (47). Folic acid during pregnancy can reduce the risk of fetal neural tube malformations, megaloblastic anemia, and pregnancy hypertension (45, 48). Wang et al. found, after adjusting for maternal weight during pregnancy, that children of mothers with adequate folate concentrations had a 43% lower risk of overweight or obesity compared to children of mothers with low folate concentrations (49). Multivitamins refer to products that are synthesized in A specific dose ratio and contain multiple vitamins including A, B, C, D, E and K. Multivitamin supplementation has been shown to effectively reduce the occurrence of complications during pregnancy, the risk of preterm birth, low birth weight, and the birth of macrosomia (50, 51).

Thanks to the extensive health education and free folic acid distribution policy in China, the utilization of nutritional supplements during pregnancy is high. The results of a 2018 survey in China showed that 82.03% of expecting mothers took nutritional supplements during pregnancy, especially with a relatively high supplementation rate of calcium and folic acid (52). Calcium supplementation is mainly due to the burden on bones caused by the weight gain of pregnant women and the demands of the fetus (46, 53). The relatively low iron supplementation rate might be due to the effectiveness of iron-containing diet intervention and the side effects of iron supplements (54, 55), but it is undeniable that iron remains one of the most commonly used nutritional supplements during pregnancy. Due to the significant increase in the demand for a variety of vitamins during pregnancy, single-vitamin supplementation may not always be sufficient to meet the nutritional needs of pregnant women. Therefore, we selected the above four nutritional supplements (iron, calcium, folic acid and multivitamins) as independent variables in this study.

The objective of this study, therefore, was to examine the combined effects of prenatal iron, calcium, folic acid and multivitamin supplementation on obesity in preschoolers born macrosomia in China. To provide a basis for the precise classification and management of obesity in the future.

## 2 Methods

### 2.1 Study population

All kindergartens in Longhua District, Shenzhen, China were surveyed for the study population, which comprised 69,639 mother-infant pairs, 7,078 of these children were macrosomia. We used the definition of macrosomia as birth weight greater than or equal to 4 kg, and the preschool age range included 3–6.5 years. Because of missing data (26 pairs due to height information, 89 pairs due to weight information, 528 pairs for mother's age at birth, 183 pairs for father's age at birth, 239 pairs for age at birth, 200 pairs for father's literacy, 157 pairs for mother's pre-pregnancy body mass index, 1 pair for monthly family income status, 1 pair for mode of delivery, and 29 pairs for maternal gestational hypertension), data from a total of 961 pairs were excluded. In addition, data from 86 pairs were excluded due to a combination of either the child or parent's age being outside the inclusion criteria (53 pairs whose children's age was higher or lower than preschool age, and 58 pairs whose parents' age was above or below the childbearing age). The final sample used in the data analysis involved 6,031 mother-child pairs of macrosomia. Written informed consent was obtained from all participants at enrollment. The study protocol was approved by the Ethics Committee of the School of Public Health, Sun Yat-sen University.

### 2.2 Data collection

Mothers who were enrolled in this study were asked to complete a self-report structured questionnaire for collecting sociodemographic characteristics of the child and parents (such as age, sex, pre-pregnancy maternal BMI, parental marital status, parental education level, household income, and whether or not the child was an only child), prenatal maternal supplementation with iron, calcium, folic acid, and multivitamins, as well as variables related to birth (mode of delivery, gestational age, and birth weight). The obtained data were verified accordingly with the data in the maternal and child health system, and the missing information during the on-site investigation was supplemented as much as possible to ensure the accuracy of the data.

### 2.3 Measurement of prenatal maternal nutrient supplementation

We collected data on iron, calcium, folic acid, and multivitamin supplementation during pregnancy using the following questions: (1) Did the mother take iron supplements during pregnancy? (2) Did mothers take calcium supplements during pregnancy? (3) Did the mother take folic acid supplements during pregnancy? (4) Did the mother take multivitamins supplements during pregnancy? The answer to each question is 1 for “yes” and 0 for “no”. In the survey, pregnant women were asked to consider whether they took nutritional supplements according to the doctor's recommendation.

### 2.4 Measurement and definition of obesity

The well-trained medical staffs from Women's and Children's Hospital of Longhua District of Shenzhen were responsible for measuring children's weight and height using a portable electronic scale (cent value = 0.01 kg) by placing the scale on a flat surface and having the preschooler stand in the center of the scale with his/her hat off, bare feet, and wearing tight-fitting lightweight clothing. After the value had stabilized, the medical staff read and recorded the measurement with an accuracy of 0.01 kg. Height was measured using a column-type body altimeter (cent value = 0.1 cm). The column-type body altimeter was placed vertically against the wall on a horizontal floor. Preschoolers were asked to stand on the pedal with heels closed, feet separated at a 60-degree angle, chest raised, abdomen tucked in, eyes looking straight ahead, and the back of the head, buttocks, and heels pressed against the column. The medical staff slid a slider to the top of the skull of the child being measured and read the measurements at a height level with the slider.

In this study, we applied the index of body mass index (BMI) as a criterion of childhood obesity diagnostic criteria, and BMI was calculated by dividing weight (kilograms) by the square of height (meters). Obesity was characterized by a BMI that fell within or exceeds the reference values for gender and age, which were derived from normative data obtained from two representative national cross-sectional surveys in China: the National 2005 Survey of Growth and Development of Children Under 7 Years of Age in Nine Cities of China and the 2005 National Monitoring of Primary and Secondary School Students' Physical Fitness and Health. Based on the above data, Li Hui et al. recommend cut-off points for obesity in Chinese children aged 2∼18 years which involved an age-specific BMI above the 96.3rd percentile for boys and the 98.0th percentile for girls (56). These cut-offs were used in the present study. A systematic review study of children and adolescents aged 2–19 years has supported the use of BMI measured by health professionals as a valid method of assessing body fat in children and adolescents (57).

### 2.5 Potential confounding variables

Based on previous findings (58, 59), potential confounding variables included in this study were the child's sex, child's age, mode of delivery, parents' age at the childbirth, maternal prepregnancy BMI, marital status, parents' education level, family income, whether the only child, gestational diabetes mellitus, patterns and duration of breastfeeding, frequency of outdoor activities at age 0–1 years, outdoor activities of time 0–1 years of age, nutritional status of 0–1 years old, frequency of outdoor activities at age 1–3 years, outdoor activities of time 1–3 years of age and nutritional status of 1–3 years old. Moreover, previous research results in our group have shown that supplementation of nutrients such as folic acid during pregnancy can affect pre-school obesity in children who are small for gestational age (60), so it is necessary to adjust other nutrients in the model.

### 2.6 Statistical analyses

Continuous variables were described by mean and standard deviation (SD), and categorical variables were described by frequency and percentage. Comparisons of categorical and numerical variables were performed using chi-square and t-tests, respectively.

A series of binary logistic regression analyses, controlling for potentially confounding variables were conducted to test the association between nutritional supplementation with iron, calcium, folic acid, and multivitamins during pregnancy and obesity in preschoolers born macrosomia. Their multiplicative interactions on obesity were tested by creating multiplicative terms in the logistic regression models, and the strength of the multiplicative interactions was expressed as the interaction of odds ratio (IOR). If the 95% CI of the IOR spanned 1, the multiplicative interaction was considered non-significant. Moreover, crossover analyses were utilized to assess the additive interaction effects and modification effects among the combination of four nutrients. Adjusted odds ratio (AOR), relative excess risk due to interaction (RERI), and attributable proportion due to interaction (AP) were calculated to quantify the magnitude of these effects. If the 95% CIs of RERI and AP did not span 0, the additive interaction was considered statistically significant. Furthermore, after stratifying by sex, the previously mentioned analyses were replicated to assess the sex-specific relationships between prenatal supplementation and obesity in preschoolers born with macrosomia.

Considering the existence of missing data, sensitivity analysis was used to compare with the results of the main analysis content to ensure the reliability of the analysis results.

RStudio version 4.1.2 (Poist, BOSTON, MA, USA) was used for all statistical analyses, and two-tailed *p*-values < 0.05 were considered statistically significant.

## 3 Results

### 3.1 Characteristics of participants

The demographic characteristics of the participants can be found in Table 1, with 62.38% of the children in the study being male and 37.62% being female. The children's mean age was 4.86 years (SD = 0.84), the father's mean age was 31.69 years (SD = 5.10), and the mother's mean age was 29.59 years (SD = 4.46). The mean birth weight of the children at birth was 4.25 kg (SD = 0.42) and the gestational week at delivery was 39.20 weeks (SD = 2.04). Vaginal deliveries (52.33%) were slightly more numerous than other modes of delivery such as cesarean section (47.67%), only 530 mothers (8.79%) admitted to a history of gestational diabetes. The mean height of the children was 111.16 cm (SD = 8.06), the mean weight was 19.83 kg (SD = 4.05), the mean BMI of the children was 15.99 kg/m^2^ (SD = 2.37), and the mean pre-pregnancy BMI of the mothers was 21.47 kg/m^2^ (SD = 3.03). Only 32.20% of the children in the study population were reported as having no siblings, and the vast majority of children had married parents (98.64%). The percentage of fathers with a high school education or above was 87.71%, while for mothers, it was 84.88%. More than three-quarters of the participants had a monthly income exceeding 10,000 CNY (86.12%). 60% of the children were exclusively breastfed after birth for an average of 9.19 months. Three quarters of children engaged in outdoor physical activity more than three times per week and more than half spent more than 60 min per time outdoors at the age of 0–1 years. More than 99 percent of parents considered their children's nutritional status to be average or above at age 0–1. By the age of 1–3 years, 69.11% of the children had outdoor exercise more than three times per week, 67.72% of the children had outdoor exercise more than one hour per time, and only 1.11% of the parents thought their children's nutritional status was poor.

**Table 1:** Comparison of demographic characteristics between obese and non-obese children born macrosomia.

Characteristics	Total	Obesity, *N* (%)		*p*
		No	Yes	
Total	6,031	5,184 (85.96)	847 (14.04)	
Age [(Mean ± SD) (years)]	4.86 ± 0.84	4.85 ± 0.85	4.89 ± 0.81	0.221
Sex				
Male	3,762 (62.38)	3,191 (61.55)	571 (67.41)	0.001
Female	2,269 (37.62)	1,993 (38.45)	276 (32.59)	
Current height of child [(Mean ± SD) (cm)]	111.16 ± 8.06	111.15 ± 7.76	111.17 ± 9.72	0.950
Current weight of child [(Mean ± SD) (kg)]	19.83 ± 4.05	18.93 ± 3.00	25.34 ± 5.11	<0.001
Current BMI of child [(Mean ± SD) (kg/m^2^)]	15.99 ± 2.37	15.27 ± 1.39	20.37 ± 2.40	<0.001
Gestational age at birth [(Mean ± SD) (weeks)]	39.20 ± 2.04	39.22 ± 2.00	39.10 ± 2.24	0.130
Birthweight [(Mean ± SD) (kg)]	4.25 ± 0.42	4.25 ± 0.42	4.25 ± 0.40	0.926
Maternal age [(Mean ± SD) (years)]	29.59 ± 4.46	29.59 ± 4.45	29.75 ± 4.52	0.255
Paternal age [(Mean ± SD) (years)]	31.69 ± 5.10	31.70 ± 5.08	31.63 ± 5.27	0.705
Maternal pre-pregnancy BMI [(Mean ± SD)	21.47 ± 3.03	21.39 ± 3.00	21.94 ± 3.20	<0.001
Mode of delivery				
Vaginal delivery	3,156 (52.33)	2,724 (52.55)	432 (51.00)	0.426
Cesarean section, forceps, vacuum extraction	2,875 (47.67)	2,460 (47.45)	415 (49.00)	
Gestational diabetes mellitus				
No	5,501 (91.21)	4,732 (91.28)	769 (90.79)	0.688
Yes	530 (8.79)	452 (8.72)	78 (9.21)	
Marital state				
Married	5,949 (98.64)	5,113 (98.63)	836 (98.70)	0.996
Divorced, remarried, spouse loss, unmarried	82 (1.36)	71 (1.37)	11 (1.30)	
The only child				
No	1,942 (32.20)	1,701 (32.81)	241 (28.45)	0.013
Yes	4,089 (67.80)	3,483 (67.19)	606 (71.55)	
Maternal education level				
Junior high school or lower	912 (15.12)	779 (15.03)	133 (15.70)	0.193
High school	1,285 (21.31)	1,087 (20.97)	198 (23.38)	
College or higher	3,834 (63.57)	3,318 (64.00)	516 (60.92)	
Paternal education level				
Junior high school or lower	741 (12.29)	638 (12.31)	103 (12.16)	0.024
High school	1,256 (20.83)	1,050 (20.25)	206 (24.32)	
College or higher	4,034 (66.89)	3,496 (67.44)	538 (63.52)	
Household income [(Chinese Yuan)]				
0–9,999	837 (13.88)	711 (13.72)	126 (14.88)	0.516
10,000–19,999	1,996 (33.10)	1,719 (33.16)	277 (32.70)	
20,000–29,999	1,292 (21.42)	1,099 (21.20)	193 (22.79)	
30,000–39,999	797 (13.22)	697 (13.45)	100 (11.81)	
≥40,000	1,109 (18.39)	958 (18.48)	151 (17.83)	
Breastfeeding patterns				
Exclusive breastfeeding	3,665 (60.77)	3,165 (61.05)	500 (59.03)	0.360
Artificial feeding	623 (10.33)	525 (10.13)	98 (11.57)	
Mixed feeding	1,743 (28.90)	1,494 (28.82)	249 (29.40)	
Breastfeeding duration [(Mean ± SD) (years)]	9.19 ± 6.47	9.23 ± 6.43	8.92 ± 6.74	0.202
Frequency of outdoor activities at age 0–1 years				
<3 times/week	1,506 (24.97)	1,272 (24.54)	234 (27.63)	0.060
≥3 times/week	4,525 (75.03)	3,912 (75.46)	613 (72.37)	
Outdoor activities of time 0–1 years of age				
<60 min/time	2,689 (44.59)	2,301 (44.39)	388 (45.81)	0.463
≥60 min/time	3,342 (55.41)	2,883 (55.61)	459 (54.19)	
Nutritional status of 0–1 years old				
Poor-nourished	57 (0.95)	50 (0.96)	7 (0.83)	0.174
General	1,170 (19.40)	1,025 (19.77)	145 (17.12)	
Well-nourished	4,804 (79.66)	4,109 (79.26)	695 (82.05)	
Frequency of outdoor activities at age 1–3 years				
<3 times/week	1,863 (30.89)	1,561 (30.11)	302 (35.66)	0.001
≥3 times/week	4,168 (69.11)	3,623 (69.89)	545 (64.34)	
Outdoor activities of time 1–3 years of age				
<60 min/time	1,947 (32.28)	1,650 (31.83)	297 (35.06)	0.068
≥60 min/time	4,084 (67.72)	3,534 (68.17)	550 (64.94)	
Nutritional status of 1–3 years old				
Poor-nourished	67 (1.11)	53 (1.02)	14 (1.65)	<0.001
General	1,561 (25.88)	1,404 (27.08)	157 (18.54)	
Well-nourished	4,403 (73.01)	3,727 (71.89)	676 (79.81)	

The prevalence of obesity in preschoolers born macrosomia was 14.04%, and the weight and BMI levels of obese children were significantly higher than those of the normal-weight population. There were significant differences in children's sex, maternal pre-pregnancy BMI, being the only child, father's education level, frequency of outdoor activities at age 1–3 years, and nutritional status of 1–3 years old.

### 3.2 Associations between the maternal nutrients supplementation during pregnancy and obesity in preschoolers born macrosomia

After controlling for potential confounding variables except nutrients, the results of logistic regression analyses showed that maternal supplementation with iron (AOR = 0.76, 95% CI = 0.65–0.90), calcium (AOR = 0.74, 95% CI = 0.61–0.91), and folic acid (AOR = 0.77, 95% CI = 0.60–0.98) during pregnancy were significantly and negatively associated with obesity in preschoolers born macrosomia. In contrast, no significant association was found between multivitamin supplementation during pregnancy and childhood obesity (Table 2). After further adjustment of other nutrients on this basis, only iron supplementation during pregnancy (AOR = 0.79, 95% CI = 0.67–0.94) was negatively associated with obesity in preschoolers born macrosomia. The adjustment process for single or two nutrients is detailed in Supplementary Tables S1, S2. Sensitivity analysis showed there was a significant effect of calcium supplementation during pregnancy on childhood obesity in girls (AOR = 0.79, 95% CI = 0.64–0.98), as shown in Supplementary Table S10.

**Table 2:** Association of maternal nutritional supplements with obesity in preschoolers born macrosomia.

Nutritional supplements	Total, *N* = 6,031	Obesity (*N*, %)	OR (95% CI)	AOR (95% CI)^a^	AOR (95% CI)^b^
Iron					
No	3,777	583 (15.44)	1.00	1.00	1.00
Yes	2,254	264 (11.71)	0.73 (0.62, 0.85)***	0.76 (0.65, 0.90)**	0.79 (0.67, 0.94)**
Calcium					
No	842	147 (17.46)	1.00	1.00	1.00
Yes	5,189	700 (13.49)	0.74 (0.61, 0.90)**	0.74 (0.61, 0.91)**	0.82 (0.66, 1.03)
Folic acid					
No	525	93 (17.71)	1.00	1.00	1.00
Yes	5,506	754 (13.69)	0.74 (0.58, 0.94)*	0.77 (0.60, 0.98)*	0.89 (0.68, 1.18)
Multivitamin					
No	3,667	541 (14.75)	1.00	1.00	1.00
Yes	2,364	306 (12.94)	0.86 (0.74, 1.00)*	0.91 (0.78, 1.07)	0.99 (0.84, 1.18)

^a^ Adjusted for child's sex, child's age, mode of delivery, parents’ age at the childbirth, maternal prepregnancy BMI, marital status, parents’ education level, family income, whether the only child, gestational diabetes mellitus, patterns and duration of breastfeeding, frequency of outdoor activities at age 0–1 years, outdoor activities of time 0–1 years of age, nutritional status of 0–1 years old, frequency of outdoor activities at age 1–3 years, outdoor activities of time 1–3 years of age and nutritional status of 1–3 years old in models.

^b^ Adjusted for child's sex, child's age, mode of delivery, parents’ age at the childbirth, maternal prepregnancy BMI, marital status, parents’ education level, family income, whether the only child, gestational diabetes mellitus, patterns and duration of breastfeeding, frequency of outdoor activities at age 0–1 years, outdoor activities of time 0–1 years of age, nutritional status of 0–1 years old, frequency of outdoor activities at age 1–3 years, outdoor activities of time 1–3 years of age, nutritional status of 1–3 years old, and other nutrients in models.

* *p* < 0.05.

** *p* < 0.01.

*** *p* < 0.001.

### 3.3 Combined effects of maternal nutrients supplementation during pregnancy on obesity in preschoolers born macrosomia

Table 3 presents the combined effects of maternal nutritional supplementation during pregnancy on obesity in preschoolers born macrosomia. After adjusting for potential confounding variables, the results of the crossover analyses showed that maternal supplementation with iron and calcium (AOR = 0.68, 95% CI = 0.52–0.88), iron and folic acid (AOR = 0.71, 95% CI = 0.51–0.98), iron and multivitamin (AOR = 0.75, 95% CI = 0.59–0.94) during pregnancy all significantly reduced the likelihood of obesity in preschoolers born macrosomia. There was a multiplication interaction effect between iron and multivitamin supplementation during pregnancy on obesity in preschoolers born macrosomia (IOR = 0.70, 95% CI = 0.50–0.97). The results of the crossover analysis adjusting separately for the other nutrient are detailed in Supplementary Table S3. Sensitivity analyses of iron supplementation showed the same results (Supplementary Table S11).

**Table 3:** Combined effects of maternal nutritional supplements on obesity in preschoolers born macrosomia.

Nutritional supplements		Total, *N* = 6,031	Obesity (*N*, %)	AOR (95% CI)^a^	AOR (95% CI)^b^	IOR (95% CI)^b^	RERI (95% CI)^b^	AP (95% CI)^b^
Iron	Calcium					0.56 (0.3, 1.04)	−0.57 (−1.41, 0.27)	−0.84 (−2.04, 0.36)
No	No	760	131 (17.24)	1.00	1.00			
No	Yes	3,015	452 (14.99)	0.84 (0.68, 1.04)	0.88 (0.69, 1.13)			
Yes	No	80	16 (20.00)	1.30 (0.72, 2.35)	1.36 (0.75, 2.48)			
Yes	Yes	2,174	248 (11.41)	0.64 (0.50, 0.81)***	0.68 (0.52, 0.88)**			
Iron	Folic acid					1.04 (0.44, 2.47)	0.06 (−0.62, 0.73)	0.08 (−0.88, 1.04)
No	No	469	86 (18.34)	1.00	1.00			
No	Yes	3,308	497 (15.02)	0.80 (0.62, 1.04)	0.89 (0.67, 1.19)			
Yes	No	56	7 (12.5)	0.69 (0.30, 1.59)	0.76 (0.33, 1.78)			
Yes	Yes	2,198	257 (11.69)	0.63 (0.47, 0.83)**	0.71 (0.51, 0.98)*			
Iron	Multivitamin					0.70 (0.50, 0.97)*	−0.66 (−0.33, 0.00)	−0.44 (−0.90, 0.01)*
No	No	2,647	407 (15.38)	1.00	1.00			
No	Yes	1,130	176 (15.58)	1.07 (0.88, 1.31)	1.14 (0.93, 1.40)			
Yes	No	1,020	134 (13.14)	0.88 (0.71, 1.09)	0.93 (0.75, 1.17)			
Yes	Yes	1,234	130 (10.53)	0.69 (0.56, 0.87)**	0.75 (0.59, 0.94)*			

^a^ Adjusted for child's sex, child's age, mode of delivery, parents’ age at the childbirth, maternal prepregnancy BMI, marital status, parents’ education level, family income, whether the only child, gestational diabetes mellitus, patterns and duration of breastfeeding, frequency of outdoor activities at age 0–1 years, outdoor activities of time 0–1 years of age, nutritional status of 0–1 years old, frequency of outdoor activities at age 1–3 years, outdoor activities of time 1–3 years of age and nutritional status of 1–3 years old in models.

^b^ Adjusted for child's sex, child's age, mode of delivery, parents’ age at the childbirth, maternal prepregnancy BMI, marital status, parents’ education level, family income, whether the only child, gestational diabetes mellitus, patterns and duration of breastfeeding, frequency of outdoor activities at age 0–1 years, outdoor activities of time 0–1 years of age, nutritional status of 0–1 years old, frequency of outdoor activities at age 1–3 years, outdoor activities of time 1–3 years of age, nutritional status of 1–3 years old, and other nutrients in models.

* *p* < 0.05.

** *p* < 0.01.

*** *p* < 0.001.

### 3.4 Combined effects of maternal nutrients supplementation during pregnancy on obesity in preschoolers born macrosomia after stratification by sex

After stratification by sex, the results of the logistic regression indicated that maternal iron supplementation (AOR = 0.75, 95% CI = 0.60–0.92) during pregnancy was significantly negatively associated with obesity in male preschoolers born macrosomia (Table 4). In girls, after controlling for the specified confounding variables except the other nutrients, the results of the logistic regression showed that maternal calcium supplementation (AOR = 0.67, 95% CI = 0.47–0.96) during pregnancy was negatively associated with the presence of obesity in female preschoolers born macrosomia. However, after adjusting for the other three nutrients, no significant results were found, especially after adjusting for folic acid (Supplementary Table S6), with the results showing the protective effect of calcium supplementation during pregnancy against preschool obesity was less significant. The adjustment process for single or two nutrients is detailed in Supplementary Tables S4–S7. Sensitivity analysis showed the same results in boys. There was a marginal effect of calcium supplementation during pregnancy on childhood obesity in girls (AOR = 0.69, 95%CI = 0.46–1.01), as shown in Supplementary Table S12.

**Table 4:** Association of maternal nutritional supplements with obesity in preschoolers born macrosomia after stratification by sex.

Sex	Nutritional supplements	Total	Obesity (*N*, %)	OR (95% CI)	AOR (95% CI)^a^	AOR (95% CI)^b^
Male						
	Iron					
	No	2,425	407 (16.78)	1.00	1.00	1.00
	Yes	1,337	164 (12.27)	0.69 (0.57, 0.84)***	0.72 (0.59, 0.88)**	0.75 (0.60, 0.92)**
	Calcium					
	No	567	101 (17.81)	1.00	1.00	1.00
	Yes	3,195	470 (14.71)	0.80 (0.63, 1.01)	0.78 (0.61, 0.99)*	0.87 (0.66, 1.14)
	Folic acid					
	No	333	61 (18.32)	1.00	1.00	1.00
	Yes	3,429	510 (14.87)	0.78 (0.59, 1.05)	0.78 (0.57, 1.06)	0.89 (0.64, 1.25)
	Multivitamin					
	No	2,314	370 (15.99)	1.00	1.00	1.00
	Yes	1,448	201 (13.88)	0.85 (0.70, 1.02)	0.89 (0.73, 1.09)	0.98 (0.80, 1.21)
Female						
	Iron					
	No	1,352	176 (13.02)	1.00	1.00	1.00
	Yes	917	100 (10.91)	0.82 (0.63, 1.06)	0.87 (0.66, 1.15)	0.92 (0.69, 1.23)
	Calcium					
	No	275	46 (16.73)	1.00	1.00	1.00
	Yes	1,994	230 (11.53)	0.65 (0.46, 0.93)*	0.67 (0.47, 0.96)*	0.71 (0.47, 1.08)
	Folic acid					
	No	192	32 (16.67)	1.00	1.00	1.00
	Yes	2,077	244 (11.75)	0.67 (0.45, 1.01)*	0.73 (0.48, 1.13)	0.88 (0.54, 1.44)
	Multivitamin					
	No	1,353	171 (12.64)	1.00	1.00	1.00
	Yes	916	105 (11.46)	0.89 (0.69, 1.16)	0.97 (0.73, 1.28)	1.05 (0.78, 1.40)

^a^ Adjusted for child's sex, child's age, mode of delivery, parents’ age at the childbirth, maternal prepregnancy BMI, marital status, parents’ education level, family income, whether the only child, gestational diabetes mellitus, patterns and duration of breastfeeding, frequency of outdoor activities at age 0–1 years, outdoor activities of time 0–1 years of age, nutritional status of 0–1 years old, frequency of outdoor activities at age 1–3 years, outdoor activities of time 1–3 years of age and nutritional status of 1–3 years old in models.

^b^ Adjusted for child's sex, child's age, mode of delivery, parents’ age at the childbirth, maternal prepregnancy BMI, marital status, parents’ education level, family income, whether the only child, gestational diabetes mellitus, patterns and duration of breastfeeding, frequency of outdoor activities at age 0–1 years, outdoor activities of time 0–1 years of age, nutritional status of 0–1 years old, frequency of outdoor activities at age 1–3 years, outdoor activities of time 1–3 years of age, nutritional status of 1–3 years old, and other nutrients in models.

* *p* < 0.05.

** *p* < 0.01.

*** *p* < 0.001.

Table 5 presents the combined effects of maternal supplementation during pregnancy on obesity in preschoolers born macrosomia after stratification by sex. Among boys, the results of the crossover analyses showed that maternal supplementation with iron and calcium (AOR = 0.68, 95% CI = 0.49–0.94), iron and multivitamin (AOR = 0.64, 95% CI = 0.48–0.86) during pregnancy significantly reduced the risk of obesity in male preschoolers born macrosomia. The results from the sensitivity analysis in boys also showed that supplementation of iron and folic acid (AOR = 0.65, 95% CI = 0.44–0.95) during pregnancy significantly reduced the risk of obesity in preschoolers born macrosomia (Supplementary Table S13). A multiplicative interaction between maternal iron and multivitamin supplementation during pregnancy on obesity was found in male preschoolers born macrosomia (IOR = 0.52, 95% CI = 0.35–0.79). Among girls, none of the cross-over analyses were significant. An additive interaction between maternal iron and folic acid supplementation during pregnancy on obesity was observed in female preschoolers born macrosomia (REFI = 0.65, 95% CI = 0.03–1.33, AP = 0.85, 95% CI = −0.18–1.88). The results of the crossover analysis adjusting separately for the other nutrient are detailed in Supplementary Table S8.

**Table 5:** Combined effects of maternal nutritional supplements on obesity in preschoolers born macrosomia after stratification by sex.

Nutritional supplements		Total	Obesity (*N*, %)	AOR (95% CI)^a^	AOR (95% CI)^b^	IOR (95% CI)^b^	RERI (95% CI)^b^	AP (95% CI)^b^
Male		3,762	571 (15.18)					
Iron	Calcium					0.46 (0.22, 0.97)*	−0.80 (−1.92, 0.31)	−1.19 (−2.77, 0.39)
No	No	511	89 (17.42)	1.00	1.00			
No	Yes	1,914	318 (16.61)	0.91 (0.70, 1.18)	0.96 (0.72, 1.28)			
Yes	No	56	12 (21.43)	1.45 (0.72, 2.91)	1.52 (0.75, 3.08)			
Yes	Yes	1,281	152 (11.87)	0.64 (0.47, 0.86)**	0.68 (0.49, 0.94)*			
Iron	Folic acid					0.68 (0.26, 1.81)	−0.33 (−1.40, 0.75)	−0.48 (−2.00, 1.04)
No	No	298	55 (18.46)	1.00	1.00			
No	Yes	2,127	352 (16.55)	0.86 (0.62, 1.19)	0.93 (0.65, 1.34)			
Yes	No	35	6 (17.14)	0.99 (0.38, 2.56)	1.08 (0.41, 2.82)			
Yes	Yes	1,302	158 (12.14)	0.62 (0.43, 0.89)**	0.69 (0.46, 1.02)			
Iron	Multivitamin					0.52 (0.35, 0.79)**	−0.59 (−1.02, −0.16)	−0.92 (−1.64, −0.19)**
No	No	1,707	279 (16.34)	1.00	1.00			
No	Yes	718	128 (17.83)	1.17 (0.92, 1.49)	1.24 (0.97, 1.59)			
Yes	No	607	91 (14.99)	0.95 (0.73, 1.23)	0.99 (0.76, 1.30)			
Yes	Yes	730	73 (10.00)	0.60 (0.45, 0.80)***	0.64 (0.48, 0.86)**			
Female		2,269	276 (12.16)					
Iron	Calcium					0.74 (0.22, 2.43)	−0.30 (−1.76, 1.15)	−0.45 (−2.59, 1.68)
No	No	251	42 (16.73)	1.00	1.00			
No	Yes	1,101	134 (12.17)	0.71 (0.48, 1.04)	0.74 (0.48, 1.14)			
Yes	No	24	4 (16.67)	1.19 (0.38, 3.75)	1.23 (0.39, 3.94)			
Yes	Yes	893	96 (10.75)	0.64 (0.42, 0.98)*	0.67 (0.42, 1.07)			
Iron	Folic acid					3.09 (0.38, 25.23)	0.65 (−0.03, 1.33)*	0.85 (−0.18, 1.88)*
No	No	171	31 (18.13)	1.00	1.00			
No	Yes	1,181	145 (12.28)	0.69 (0.44, 1.08)	0.81 (0.49, 1.34)			
Yes	No	21	1 (4.76)	0.27 (0.03, 2.10)	0.31 (0.04, 2.47)			
Yes	Yes	896	99 (11.05)	0.64 (0.40, 1.03)	0.77 (0.44, 1.34)			
Iron	Multivitamin					1.12 (0.64, 1.98)	0.11 (−0.42, 0.64)	0.11 (−0.43, 0.65)
No	No	940	128 (13.62)	1.00	1.00			
No	Yes	412	48 (11.65)	0.92 (0.64, 1.33)	1.00 (0.68, 1.46)			
Yes	No	413	43 (10.41)	0.80 (0.55, 1.17)	0.87 (0.59, 1.29)			
Yes	Yes	504	57 (11.31)	0.89 (0.62, 1.28)	0.98 (0.67, 1.43)			

^a^ Adjusted for child's sex, child's age, mode of delivery, parents’ age at the childbirth, maternal prepregnancy BMI, marital status, parents’ education level, family income, whether the only child, gestational diabetes mellitus, patterns and duration of breastfeeding, frequency of outdoor activities at age 0–1 years, outdoor activities of time 0–1 years of age, nutritional status of 0–1 years old, frequency of outdoor activities at age 1–3 years, outdoor activities of time 1–3 years of age and nutritional status of 1–3 years old in models.

^b^ Adjusted for child's sex, child's age, mode of delivery, parents’ age at the childbirth, maternal prepregnancy BMI, marital status, parents’ education level, family income, whether the only child, gestational diabetes mellitus, patterns and duration of breastfeeding, frequency of outdoor activities at age 0–1 years, outdoor activities of time 0–1 years of age, nutritional status of 0–1 years old, frequency of outdoor activities at age 1–3 years, outdoor activities of time 1–3 years of age, nutritional status of 1–3 years old, and other nutrients in models.

* *p* < 0.05.

** *p* < 0.01.

*** *p* < 0.001.

## 4 Discussion

To our knowledge, this is the first study to examine the combined effects of maternal supplementation with iron, calcium, folic acid, and multivitamin during pregnancy on obesity in preschoolers born macrosomia in China. The results of supplementary correlation tests showed that the ingestion of any two nutrients were not completely independent, but the correlation coefficients were all small (Supplementary Table S9).

### 4.1 Associations between the maternal iron supplementation during pregnancy and obesity in preschoolers born macrosomia

Iron supplementation during pregnancy was found to be significantly associated with a reduced risk of obesity in preschoolers born with macrosomia. However, this result was only found in boys. Previous studies on the relationship between maternal iron supplementation during pregnancy have mostly focused on birth weight and the presence of obesity in adulthood, with few studies examining the impact upon obesity during the early childhood period. The Cambridge Baby Growth Study found that at 3 months of age, infants from high-income families, whose mothers had received iron supplementation during pregnancy, had 0.15 mm thicker skinfold than those whose mothers had not received iron supplementation. However, no such differences in obesity indicators were found in subsequent follow-up at 2 years and 9.5 years of age (61). Similarly, previous results from another birth cohort in the United Kingdom did not find an association between maternal iron intake during pregnancy and obesity in children at age 10 years (62). Studies using C282Y of the hemochromatosis gene (*HFE*) as an instrumental variable of iron status also failed to find an association between iron during pregnancy and obesity in adult offspring (63). The differences in the above results may be due to the differences in different ethnic populations, the ages of the research subjects, and the corresponding obesity screening indicators.

However, the results of experiments with rats show that iron supplementation during pregnancy can reduce plasma triglyceride levels in fetus and adult rats, and reduce the risk of metabolic diseases such as obesity by down-regulating the expression of genes involved in bile acid synthesis and fatty acid synthesis pathways, especially cholesterol 7α-hydroxylase and its upstream regulator liver X receptor *α* (44). Perinatal iron deficiency in Dawley rats increases the effect of high-fat diet on offspring body weight, and this body weight gain is mainly due to the accumulation of visceral fat (64). Hepcidin is a major inhibitor of intestinal iron absorption. Emanuele et al. found that hepcidin concentration was significantly increased in obese children and showed a clear dose-response relationship (65). Maternal pre-pregnancy BMI, iron supplementation, erythropoiesis-stimulating activity and inflammatory response all affect maternal hepcidin secretion (66). Nicole et al. found that the amount of placental transfer of maternal iron and neonatal iron absorption were related to the levels of inflammatory factors. Although the study did not directly observe the correlation with maternal hepcidin, this may be due to the relative intensity of maternal inflammation and iron depletion, which offset each other (67, 68). Given that iron is highly abundant in the placenta and is an essential cofactor for the formation of reactive oxygen species (ROS), excessive iron accumulation can lead to excessive ROS production and severe autophagy defects in the offspring (69, 70). At the same time, mitochondrial function defects and mitochondrial DNA damage caused by iron deficiency also increase ROS formation (71). The essence of obesity is a systemic chronic inflammatory response. Changes in iron content during pregnancy lead to changes in inflammatory response and oxidative status in offspring, resulting in metabolic or functional disorders, which in turn leads to increased susceptibility to obesity and its related complications (72). These biological mechanisms support our finding, and suggest that the impact of ingesting iron supplements during pregnancy on macrosomia may involve long-term programming effects.

Why did such results only occur in male children? *In vitro* experiments have found that apoptosis caused by iron-dependent lipid peroxidation induced by the transcription factor BACH1 can stimulate the secretion of FGF21 and inhibit obesity in mice on a high-fat diet (73). A study in the United States found dietary iron deficiency deregulates iron balance in the inguinal White adipose tissue (iWAT) and impairs adaptive thermogenesis, thereby escalating the diet-induced weight gain in male mice (74). An animal experiment in Japan found that iron supplementation regulates obesity and hepatic steatosis induced by a high-fat diet in male mice through mitochondrial signaling (75).A study in Beijing involving school-aged children found that epigenetic modifications of nuclear DNA and mitochondrial DNA were associated with the disorder of serum iron biomarkers in metabolically unhealthy obese children (76). Most of the discussions on the mechanism of iron on male obesity have focused on dietary induction after birth, but the mechanism also provides enlightenment on the effect of maternal iron dose on offspring.

### 4.2 Combined effects of maternal nutrients supplementation during pregnancy on obesity in preschoolers born macrosomia after stratification

In male macrosomia, we also found that there was a multiplicative interaction effect of maternal iron supplementation and multivitamin supplementation on preschool obesity, but no significant independent effect of maternal multivitamin supplementation on preschool obesity in male. Multivitamin supplementation during pregnancy could enhance the protective effect of iron supplementation on preschool obesity in boys born with macrosomia. In contrast to our findings, several studies in North American laboratories have shown that a high-multivitamin diet during pregnancy increases the development of an obese phenotype in male offspring, involving gut microbiota abundance and epigenetic changes in hypothalamic systems that regulate food intake. The high-vitamin diet group had higher leptin concentrations and leptin receptor expression and decreased proopiomelanocortin (POMC) expression related to appetite suppression (77–79). Multivitamin supplementation during pregnancy may have an obesogenic effect on the offspring Unfortunately, there are no studies on the mechanism of the combined effects of iron and multivitamins supplenmentation during pregnancy. As mentioned earlier, animal experiments have shown that rats with perinatal iron deficiency have increased visceral fat volume (64). In a mother-infant cohort study in Singapore, it was observed that maternal vitamin D levels in the second trimester of pregnancy were negatively correlated with neonatal abdominal subcutaneous adipose tissue volume, especially deep subcutaneous adipose tissue (DSAT), whose metabolism is similar to visceral adipose tissue (80). It is speculated that iron and vitamin D may interact at the binding sites of fat metabolism-related receptors and thus affect the outcome of obesity. However, the mechanisms and pathways of various nutrients on obesity are extremely complex, and further animal experiments and human trials are needed to prove.

The protective effect of iron supplementation on preschool obesity in boys born with macrosomia appeared to be enhanced by incorporating calcium supplementation simultaneously. No research has been conducted on the effects of ingesting iron and calcium supplements simultaneously during pregnancy on childhood obesity. After supplementation, calcium enters the body and is converted into calcium ions (Ca^2+^). As a second messenger, it mediates multiple non-selective cation channel family Transient Receptor Potential (TRP) channels in adipocytes, regulates the differentiation and maturation of white adipose tissue and the formation of brown adipose tissue (81). The novel tetraspanin MS4A15 interacts with Ca^2+^ to specifically block ferroptosis by altering the lipid profile of overexpressing cells (82). Iron and calcium can jointly regulate mitophagy and cell metabolism to affect homeostasis. Combined, this evidence supports our finding that prenatal iron and calcium supplementation may have a protective effect against offspring's obesity.

In female children with macrosomia, the results showed there was an additive interaction between folic acid and iron supplementation during pregnancy on preschool childhood obesity, but folic acid and iron supplementation had no significant association with preschool obesity. Therefore, the results should be interpreted with caution. However, in contrast a randomized controlled trial in China found no difference between iron-folic acid supplementation and iron supplementation alone (83). The Boston Birth cohort found a nonlinear L-shaped association between maternal plasma folate concentration in the third trimester and the risk of offspring overweight and obesity (mean age, 6.2 years) (49). The results of a systematic review has also supports that folic acid supplementation during pregnancy had a protective effect on offspring obesity and insulin resistance (84). Similarly, both iron and folic acid supplementation during pregnancy can reduce the risk of adverse pregnancy outcomes such as preterm birth and stillbirth and the mortality of offspring within 5 years of age (85, 86). In a previous study of small for gestational age infants, our research group found that iron supplementation during pregnancy enhanced the protective effect of folic acid supplementation during pregnancy on childhood obesity, but no significant interaction was found (60). As a methyl donor of one-carbon metabolism, folate participates in epigenetic regulation such as DNA methylation, DNA synthesis and repair, and plays an important role in normal fetal growth and development (87). Both *in vitro* and in mice, folic acid supplementation within the physiological range has been found to induce differential expression of growth genes in placental cells, thereby affecting fetal weight (88, 89). Animal experiments suggest that iron metabolism regulates folate-dependent one-carbon unit cycle metabolism (90), and supplementation of iron and folic acid can promote the transcription of genes encoding folate and ferroportin (91). The literature is still unclear regarding the interaction between iron and folate. For example, on the one hand Joanna et al. found that the combination of iron and folic acid significantly reduced iron levels in the liver and spleen of female rats (92). So far, no studies, outside our research group, have examined the combined effects of the two on obesity.

### 4.3 Sensitivity analyses

We excluded information from 1,047 mother-infant pairs before analysis. After excluding height, weight, and nutritional supplement missing data, there were 6,936 mother-infant pairs. In the sensitivity analyses based on this population with missing data, similar significant results of iron supplementation on preschool obesity in male macrosomia were found (Supplementary Tables S10–S13). However, in the sensitivity analysis among girls, the results of calcium supplementation showed a significant protective effect against preschool obesity in macrosomia, whereas only a marginal effect was found in our main analyses. In addition, we found that iron and folic acid supplementation during pregnancy could enhance the protective effect of calcium supplementation on preschool obesity in girls born with macrosomia. This indicates that the removal of missing information has a certain bias on the results, which affects the significance of some results. Based on 15 randomized controlled trials worldwide, a meta-analysis revealed that calcium supplementation (500–2,000 mg) can increase fetal birth weight (93). However, in contrast an exploratory analysis carried out by Karakis et al., calcium intake was not found to be related to obesity in children aged 5 years and above (94). This result may be due to the differences in the period, dosage and continuity of calcium supplementation during pregnancy. It has been found in obesity-prone rats that low calcium can lead to an increase in body fat and a decrease in body protein, thereby affecting early body weight (95). Li et al. found that abnormal calcium intake during pregnancy and lactation, such as calcium deficiency, low calcium, and high calcium, may increase adipocytes by encoding the adipogenic differentiation potential of offspring bone marrow mesenchymal stem cells and regulating the significant expression of Wnt/β-catenin signaling pathway, which aggravates obesity induced by high-fat diet in adulthood (96). Unfortunately, these results are based only on male offspring.

### 4.4 Sex specific differences

As described above, the relationship between supplementation and combined effects of different nutrients during pregnancy and preschool obesity in macrosomia differs between males and females. We propose that there may be a number of possible reasons for sex being a moderator in these associations. First, it is possible that this may be due sex differences in body fat composition, a difference that has been observed as early as the third trimester, suggesting that mesenchymal cells may have sex differences in determining the fate of muscle and adipocyte lineages (97). Second, experiments, by Gallou-Kabani et al., in mice showed that the overall methylation pattern of the placenta was different between males and females, even when they were exposed to the same uterine environment (98). Third, boys develop more rapidly *in utero* than girls, and boys have longer placenta but lower reserve capacity, increasing their vulnerability to malnutrition (99). Fourth, sex differences in hypothalamic gene expression of pro-melanocortin, leptin receptor (genes that inhibits food intake) and neuropeptide Y, agouti associated protein (genes that promotes food intake), which are involved in the regulation of food intake, were found in the rat study (100).

### 4.5 Limitation

The results of this study have some limitations as follows. First, this study is a retrospective observational study with all data about the use of nutritional supplementation coming from self-report of mothers 3–6.5 years post-birth. As such, their recall might have been be affected by memory bias. Second, the present study was qualitative and didn't collect information on the dose, frequency, and duration of nutrient use, or the serum levels of the nutrients investigated in this study. In addition to including these measures, in future studies, researchers should consider measuring biomarkers such as the nutritional concentrations in the mothers' or umbilical cord blood to validate self-reported supplementation and obtain quantitative information. Due to the lack of this quantitative information, the conclusions we draw are preliminary and need to be replicated using more precise measures of nutrient supplementation. This is a complicated issue that requires further validation studies given that the timing, dosage and duration of nutrient supplementation recommended by Chinese doctors is personalized for each mother and changes over the course of the pregnancy. Third, the study participants were all from Longhua District, Shenzhen, China. There may be some limitations in extrapolating the results from this sample to other populations due to differences in diet. Fourth, we only evaluated the effect of nutritional supplementation during pregnancy and did not measure maternal nutrient intake through diet during pregnancy. Future studies may also include the use of food frequency questionnaires to derive more accurate associations. Fifth, we used BMI as a measure of obesity. The index of BMI shows obvious racial and regional differences. The screening criteria adopted in this study were different from those of WHO and IOTF. Therefore, it will be important to replicate the findings of this study using other measures of obesity such as skinfold thickness and waist circumference, or utilizing bioelectrical impedance or dual-energy x-ray absorptiometry as measures of body fat. Sixth, obesity is a complicated disease caused by multiple factors, including genetic and behavioral factors, and the confounding factors adjusted for in this study wasn't exhaustive. Other potential covariates need to include in future studies such as paternal obesity, maternal diet during pregnancy and the dietary nutrition of infants or preschoolers. Seventh, we excluded information from 1,047 (14.79%) mother-infant pairs that could have caused selection bias before analysis. Eighth, we did not collect the information on the specific vitamins that comprised the multivitamin used by the mothers during pregnancy, so we could not identify the associations between specific combinations of vitamin supplementation and offspring' obesity.

## 5 Conclusions

To summarize, our study demonstrates that maternal iron supplementation during pregnancy significantly affects the likelihood of obesity in male preschoolers born with macrosomia. Calcium and multivitamin supplementation during pregnancy could enhance the protective effect of iron supplementation on obesity in male macrosomia. These findings suggest that encouraging maternal iron supplementation during pregnancy may reduce the risk of obesity in male preschoolers born macrosomia. However, these conclusions may be limited by the lack of more detailed quantitative data about the nutrient supplementation engaged in by the participants. Further research is required to explore how timing, dosage and duration of maternal nutrient supplementation during pregnancy may predict childhood obesity in children with macrosomia, and clarify how sex may be a moderator of these associations.

## Data Availability

The raw data supporting the conclusions of this article will be made available by the authors, without undue reservation.
