# Supplementary material for: Combined effects of maternal supplementation of iron, calcium, folic acid, and multivitamin during pregnancy on obesity in Chinese preschoolers born macrosomia
Source: Front Pediatr. 2025 Jun 27;13:1608521. doi: 10.3389/fped.2025.1608521 (PMC12245912; doi:10.3389/fped.2025.1608521)
Supplement: Supplementary file 1 [file Datasheet1.pdf]

Supplementary Material

Table S1: Model adjustment for maternal nutrients supplementation during pregnancy and obesity in preschoolers born macrosomia (adjustment for only one nutrient or the other three nutrients).

Nutrients Supplementation	AOR (95% CI) [a]	AOR (95% CI) [b]	AOR (95% CI) [c]	AOR (95% CI) [d]	AOR (95% CI) [e]	AOR (95% CI) [f]
Iron						
No	1.00	/	1.00	1.00	1.00	1.00
Yes	0.76 (0.65, 0.90)**	/	0.79 (0.67, 0.93)**	0.78 (0.66, 0.91)**	0.77 (0.65, 0.91)**	0.79 (0.67, 0.94)**
Calcium						
No	1.00	1.00	/	1.00	1.00	1.00
Yes	0.74 (0.61, 0.91)**	0.79 (0.65, 0.97)*	/	0.78 (0.62, 0.97)*	0.75 (0.61, 0.93)**	0.82 (0.66, 1.03)
Folic acid						
No	1.00	1.00	1.00	/	1.00	1.00
Yes	0.77 (0.60, 0.98)*	0.81 (0.63, 1.04)	0.87 (0.66, 1.15)	/	0.78 (0.61, 1.01)	0.89 (0.68, 1.18)
Multivitamin						
No	1.00	1.00	1.00	1.00	/	1.00
Yes	0.91 (0.78, 1.07)	0.96 (0.81, 1.13）	0.95 (0.81, 1.12)	0.93 (0.79, 1.10)	/	0.99 (0.84, 1.18)

[a]: model 1 adjusted for child's sex, child's age, mode of delivery, parents' age at the childbirth, maternal prepregnancy BMI, marital status, parents' education level, family income, whether the only child, gestational diabetes mellitus, patterns and duration of breastfeeding, frequency of outdoor activities at age 0-1 years, outdoor activities of time 0-1 years of age, nutritional status of 0-1 years old, frequency of outdoor activities at age 1-3 years, outdoor activities of time 1-3 years of age and nutritional status of 1-3 years old in models.

[b]: model 2 adjusted for variables in model 1 and iron supplementation.

[c]: model 3 adjusted for variables in model 1 and calcium supplementation.

[d]: model 4 adjusted for variables in model 1 and folic acid supplementation.

[e]: model 5 adjusted for variables in model 1 and multivitamin supplementation.

[f]: model 6 adjusted for variables in model 1 and other three nutrients supplementation.

* $p < 0.05$, ** $p < 0.01$, *** $p < 0.001$.

Table S2: Model adjustment for maternal nutrients supplementation during pregnancy and obesity in preschoolers born macrosomia (adjustment for two nutrients).

**Nutrients Supplementation**	**AOR (95% CI)** [a]	**AOR (95% CI)** [b]	**AOR (95% CI)** [c]	**AOR (95% CI)** [d]	**AOR (95% CI)** [e]	**AOR (95% CI)** [f]	**AOR (95% CI)** [g]
Iron							
No	1.00	/	/	1.00	/	1.00	1.00
Yes	0.76 (0.65, 0.90)**	/	/	0.79 (0.67, 0.94)**	/	0.79 (0.67, 0.94)**	0.78 (0.66, 0.92)**
Calcium							
No	1.00	/	1.00	/	1.00	/	1.00
Yes	0.74 (0.61, 0.91)**	/	0.82 (0.66, 1.03)	/	0.79 (0.64, 0.98)*	/	0.79 (0.63, 0.98)*
Folic acid							
No	1.00	1.00	/	/	1.00	1.00	/
Yes	0.77 (0.60, 0.98)*	0.89 (0.68, 1.17)	/	/	0.82 (0.63,1.05)	0.88 (0.67, 1.16)	/
Multivitamin							
No	1.00	1.00	1.00	1.00	/	/	/
Yes	0.91 (0.78, 1.07)	0.99 (0.84, 1.17)	0.98 (0.83, 1.15)	0.96 (0.84, 1.13)	/	/	/

[a]: model 1 adjusted for child's sex, child's age, mode of delivery, parents' age at the childbirth, maternal prepregnancy BMI, marital status, parents' education level, family income, whether the only child, gestational diabetes mellitus, patterns and duration of breastfeeding, frequency of outdoor activities at age 0-1 years, outdoor activities of time 0-1 years of age, nutritional status of 0-1 years old, frequency of outdoor activities at age 1-3 years, outdoor activities of time 1-3 years of age and nutritional status of 1-3 years old in models.

[b]: model 2 adjusted for variables in model 1, iron and calcium supplementation.

[c]: model 3 adjusted for variables in model 1, iron and folic acid supplementation.

[d]: model 4 adjusted for variables in model 1, calcium and folic acid supplementation.

[e]: model 5 adjusted for variables in model 1, iron and multivitamin supplementation.

[f]: model 6 adjusted for variables in model 1, calcium and multivitamin supplementation.

[g]: model 7 adjusted for variables in model 1, folic acid and multivitamin supplementation.

$^{*}p < 0.05$, $^{**}p < 0.01$, $^{***}p < 0.001$.

Table S3: Model adjustment for combined effects of maternal nutrients supplementation on obesity in preschoolers born macrosomia.

**Nutrients Supplementation**		**AOR (95% CI) [a]**	**AOR (95% CI) [b]**	**AOR (95% CI) [c]**	**AOR (95% CI) [d]**
Iron	*Calcium*				
NO	NO	1.00	1.00	1.00	1.00
NO	YES	0.84 (0.68, 1.04)	0.88 (0.70, 1.12)	0.84 (0.67, 1.05)	0.88 (0.69, 1.13)
YES	NO	1.30 (0.72, 2.35)	1.36 (0.75, 2.48)	1.30 (0.72, 2.35)	1.36 (0.75, 2.48)
YES	YES	0.64 (0.50, 0.81)***	0.68 (0.52, 0.88)**	0.64 (0.50, 0.82)***	0.68 (0.52, 0.88)**
Iron	*Folic acid*				
NO	NO	1.00	1.00	1.00	1.00
NO	YES	0.80 (0.62, 1.04)	0.89 (0.67, 1.19)	0.81 (0.62, 1.05)	0.89 (0.67, 1.19)
YES	NO	0.69 (0.30, 1.59)	0.76 (0.33, 1.78)	0.69 (0.30, 1.59)	0.76 (0.33, 1.78)
YES	YES	0.63 (0.47, 0.83)**	0.71 (0.51, 0.97)*	0.63 (0.47, 0.85)**	0.71 (0.51, 0.98)*
Iron	*Multivitamin*				
NO	NO	1.00	1.00	1.00	1.00
NO	YES	1.07 (0.88, 1.31)	1.13 (0.92, 1.38)	1.11 (0.91, 1.36)	1.14 (0.93, 1.40)
YES	NO	0.88 (0.71, 1.09)	0.93 (0.74, 1.16)	0.91 (0.73, 1.13)	0.93 (0.75, 1.17)
YES	YES	0.69 (0.56, 0.87)**	0.74 (0.59, 0.93)**	0.72 (0.57, 0.90)**	0.75 (0.59, 0.94)*

[a]: model 1 adjusted for child's sex, child's age, mode of delivery, parents' age at the childbirth, maternal prepregnancy BMI, marital status, parents' education level, family income, whether the only child, gestational diabetes mellitus, patterns and duration of breastfeeding, frequency of outdoor activities at age 0-1 years, outdoor activities of time 0-1 years of age, nutritional status of 0-1 years old, frequency of outdoor activities at age 1-3 years, outdoor activities of time 1-3 years of age and nutritional status of 1-3 years old in models.

[b]: model 2 adjusted for variables in model 1 and one other nutrient in addition to these two. The specific types of nutrients adjusted from top to bottom were: combined effect of iron and calcium adjusted folic acid; combined effects of iron and folic acid adjusted calcium; combined effects of calcium and folic acid adjusted iron; combined effect of iron and multivitamin adjusted calcium; combined effect of calcium and multivitamin adjusted iron; combined effect of folic acid and multivitamin adjusted iron;

[c]: model 3 adjusted for variables in model 1 and the other nutrient that was not adjusted in model 2. The specific types of nutrients adjusted from top to bottom were: combined effect of iron and calcium adjusted multivitamin; combined effects of iron and folic acid adjusted multivitamin; combined effects of calcium and folic acid adjusted multivitamin; combined effect of iron and multivitamin adjusted folic acid; combined effect of calcium and multivitamin adjusted folic acid; combined effect of folic acid and multivitamin adjusted calcium;

[d]: model 4 adjusted for variables in model 1 and the other two nutrients.

Table S4: Model adjustment for maternal nutrients supplementation during pregnancy and obesity in preschoolers born macrosomia after stratification by sex in male (adjustment for only one nutrient or the other three nutrients).

**Nutrients Supplementation**	**AOR (95% CI) [a]**	**AOR (95% CI) [b]**	**AOR (95% CI) [c]**	**AOR (95% CI) [d]**	**AOR (95% CI) [e]**	**AOR (95% CI) [f]**
Iron						
No	1.00	/	1.00	1.00	1.00	1.00
Yes	0.72 (0.59, 0.88)**	/	0.74 (0.60, 0.91)**	0.73 (0.60, 0.90)**	0.73 (0.59, 0.89)**	0.75 (0.60, 0.92)**
Calcium						
No	1.00	1.00	/	1.00	1.00	1.00
Yes	0.78 (0.61, 0.99)*	0.84 (0.65, 1.07)	/	0.82 (0.62, 1.06)	0.79 (0.62, 1.02)	0.87 (0.66, 1.14)
Folic acid						
No	1.00	1.00	1.00	/	1.00	1.00
Yes	0.78 (0.57, 1.06)	0.83 (0.61, 1.13)	0.87 (0.62, 1.21)	/	0.80 (0.58, 1.09)	0.89 (0.64, 1.25)
Multivitamin						
No	1.00	1.00	1.00	1.00	/	1.00
Yes	0.89 (0.73, 1.09)	0.95 (0.78, 1.16)	0.93 (0.76, 1.14)	0.92 (0.75, 1.12)	/	0.98 (0.80, 1.21)

[a]: model 1 adjusted for child's age, mode of delivery, parents' age at the childbirth, maternal prepregnancy BMI, marital status, parents' education level, family income, whether the only child, gestational diabetes mellitus, patterns and duration of breastfeeding, frequency of outdoor activities at age 0-1 years, outdoor activities of time 0-1 years of age, nutritional status of 0-1 years old, frequency of outdoor activities at age 1-3 years, outdoor activities of time 1-3 years of age and nutritional status of 1-3 years old in models.

[b]: model 2 adjusted for variables in model 1 and iron supplementation.

[c]: model 3 adjusted for variables in model 1 and calcium supplementation.

[d]: model 4 adjusted for variables in model 1 and folic acid supplementation.

[e]: model 5 adjusted for variables in model 1 and multivitamin supplementation.

[f]: model 6 adjusted for variables in model 1 and other three nutrients supplementation.

** $p < 0.01$, *** $p < 0.001$.

Table S5: Model adjustment for maternal nutrients supplementation during pregnancy and obesity in preschoolers born macrosomia after stratification by sex in male (adjustment for two nutrients).

Nutrients Supplementation	AOR (95% CI) [a]	AOR (95% CI) [b]	AOR (95% CI) [c]	AOR (95% CI) [d]	AOR (95% CI) [e]	AOR (95% CI) [f]	AOR (95% CI) [g]
Iron							
No	1.00	/	/	1.00	/	1.00	1.00
Yes	0.72 (0.59, 0.88)**	/	/	0.74 (0.60, 0.91)**	/	0.75 (0.60, 0.92)**	0.73 (0.60, 0.90)**
Calcium							
No	1.00	/	1.00	/	1.00	/	1.00
Yes	0.78 (0.61, 0.99)*	/	0.87 (0.66, 1.14)	/	0.84 (0.65, 1.09)	/	0.82 (0.63, 1.08)
Folic acid							
No	1.00	1.00	/	/	1.00	1.00	/
Yes	0.78 (0.57, 1.06)	0.89 (0.64, 1.25)	/	/	0.84 (0.61,1.15)	0.87 (0.62, 1.23)	/
Multivitamin							
No	1.00	1.00	1.00	1.00	/	/	/
Yes	0.89 (0.73, 1.09)	0.98 (0.80, 1.20)	0.97 (0.79, 1.19)	0.94 (0.77, 1.15)	/	/	/

[a]: model 1 adjusted for child's age, mode of delivery, parents' age at the childbirth, maternal prepregnancy BMI, marital status, parents' education level, family income, whether the only child, gestational diabetes mellitus, patterns and duration of breastfeeding, frequency of outdoor activities at age 0-1 years, outdoor activities of time 0-1 years of age, nutritional status of 0-1 years old, frequency of outdoor activities at age 1-3 years, outdoor activities of time 1-3 years of age and nutritional status of 1-3 years old in models.

[b]: model 2 adjusted for variables in model 1, iron and calcium supplementation.

[c]: model 3 adjusted for variables in model 1, iron and folic acid supplementation.

[d]: model 4 adjusted for variables in model 1, calcium and folic acid supplementation.

[e]: model 5 adjusted for variables in model 1, iron and multivitamin supplementation.

[f]: model 6 adjusted for variables in model 1, calcium and multivitamin supplementation.

[g]: model 7 adjusted for variables in model 1, folic acid and multivitamin supplementation.

** $p < 0.01$, *** $p < 0.001$.

Table S6: Model adjustment for maternal nutrients supplementation during pregnancy and obesity in preschoolers born macrosomia after stratification by sex in female (adjustment for only one nutrient or the other three nutrients).

Nutrients Supplementation	AOR (95% CI) [a]	AOR (95% CI) [b]	AOR (95% CI) [c]	AOR (95% CI) [d]	AOR (95% CI) [e]	AOR (95% CI) [f]
Iron						
No	1.00	/	1.00	1.00	1.00	1.00
Yes	0.87 (0.66, 1.15)	/	0.92 (0.70, 1.23)	0.89 (0.68, 1.18)	0.87 (0.66, 1.15)	0.92 (0.69, 1.23)
Calcium						
No	1.00	1.00	/	1.00	1.00	1.00
Yes	0.67 (0.47, 0.96)*	0.69 (0.48, 0.99)*	/	0.70 (0.55, 1.05)	0.67 (0.46, 0.96)*	0.71 (0.47, 1.08)
Folic acid						
No	1.00	1.00	1.00	/	1.00	1.00
Yes	0.73 (0.48, 1.13)	0.75 (0.49, 1.16)	0.88 (0.55, 1.43)	/	0.73 (0.47, 1.13)	0.88 (0.54, 1.44)
Multivitamin						
No	1.00	1.00	1.00	1.00	/	1.00
Yes	0.97 (0.73, 1.28)	0.99 (0.75, 1.32)	1.03 (0.77, 1.37)	1.00 (0.75, 1.33)	/	1.05 (0.78, 1.40)

[a]: model 1 adjusted for child's age, mode of delivery, parents' age at the childbirth, maternal prepregnancy BMI, marital status, parents' education level, family income, whether the only child, gestational diabetes mellitus, patterns and duration of breastfeeding, frequency of outdoor activities at age 0-1 years, outdoor activities of time 0-1 years of age, nutritional status of 0-1 years old, frequency of outdoor activities at age 1-3 years, outdoor activities of time 1-3 years of age and nutritional status of 1-3 years old in models.

[b]: model 2 adjusted for variables in model 1 and iron supplementation.

[c]: model 3 adjusted for variables in model 1 and calcium supplementation.

[d]: model 4 adjusted for variables in model 1 and folic acid supplementation.

[e]: model 5 adjusted for variables in model 1 and multivitamin supplementation.

[f]: model 6 adjusted for variables in model 1 and other three nutrients supplementation.

* $p < 0.05$.

Table S7: Model adjustment for maternal nutrients supplementation during pregnancy and obesity in preschoolers born macrosomia after stratification by sex in female (adjustment for two nutrients).

Nutrients Supplementation	AOR (95% CI) [a]	AOR (95% CI) [b]	AOR (95% CI) [c]	AOR (95% CI) [d]	AOR (95% CI) [e]	AOR (95% CI) [f]	AOR (95% CI) [g]
Iron							
No	1.00	/	/	1.00	/	1.00	1.00
Yes	0.87 (0.66, 1.15)	/	/	0.93 (0.70, 1.24)	/	0.92 (0.69, 1.23)	0.89 (0.67, 1.18)
Calcium							
No	1.00	/	1.00	/	1.00	/	1.00
Yes	0.67 (0.47, 0.96)*	/	0.72 (0.48, 1.08)	/	0.68 (0.47, 0.99)*	/	0.70 (0.46, 1.05)
Folic acid							
No	1.00	1.00	/	/	1.00	1.00	/
Yes	0.73 (0.48, 1.13)	0.89 (0.55, 1.44)	/	/	0.75 (0.48, 1.17)	0.88 (0.54, 1.43)	/
Multivitamin							
No	1.00	1.00	1.00	1.00	/	/	/
Yes	0.97 (0.73, 1.28)	1.04 (0.78, 1.39)	1.02 (0.77, 1.36)	1.04 (0.78, 1.38)	/	/	/

[a]: model 1 adjusted for child's age, mode of delivery, parents' age at the childbirth, maternal prepregnancy BMI, marital status, parents' education level, family income, whether the only child, gestational diabetes mellitus, patterns and duration of breastfeeding, frequency of outdoor activities at age 0-1 years, outdoor activities of time 0-1 years of age, nutritional status of 0-1 years old, frequency of outdoor activities at age 1-3 years, outdoor activities of time 1-3 years of age and nutritional status of 1-3 years old in models.

[b]: model 2 adjusted for variables in model 1, iron and calcium supplementation.

[c]: model 3 adjusted for variables in model 1, iron and folic acid supplementation.

[d]: model 4 adjusted for variables in model 1, calcium and folic acid supplementation.

[e]: model 5 adjusted for variables in model 1, iron and multivitamin supplementation.

[f]: model 6 adjusted for variables in model 1, calcium and multivitamin supplementation.

[g]: model 7 adjusted for variables in model 1, folic acid and multivitamin supplementation.

* $p < 0.05$.

Table S8: Model adjustment for combined effects of maternal nutrients supplementation on obesity in preschoolers born macrosomia after stratification by sex.

**Sex**	**Nutrients Supplementation**		***AOR* (95% *CI*) [a]**	**AOR (95% CI) [b]**	**AOR (95% CI) [c]**	***AOR* (95% *CI*) [d]**
Male						
	Iron	*Calcium*				
	NO	NO	1.00	1.00	1.00	1.00
	NO	YES	0.91 (0.70, 1.18)	0.96 (0.72, 1.28)	0.91 (0.70, 1.19)	0.96 (0.72, 1.28)
	YES	NO	1.45 (0.72, 2.91)	1.52 (0.75, 3.08)	1.45 (0.72, 2.91)	1.52 (0.75, 3.08)
	YES	YES	0.64 (0.47, 0.86)**	0.67 (0.49, 0.93)*	0.64 (0.47, 0.87)**	0.68 (0.49, 0.94)*
	Iron	*Folic acid*				
	NO	NO	1.00	1.00	1.00	1.00
	NO	YES	0.86 (0.62, 1.19)	0.93 (0.65, 1.33)	0.87 (0.62, 1.21)	0.93 (0.65, 1.34)
	YES	NO	0.99 (0.38, 2.56)	1.08 (0.41, 2.82)	0.99 (0.38, 2.57)	1.08 (0.41, 2.82)
	YES	YES	0.62 (0.43, 0.89)**	0.68 (0.46, 1.02)	0.63 (0.44, 0.91)*	0.69 (0.46, 1.02)
	Iron	*Multivitamin*				
	NO	NO	1.00	1.00	1.00	1.00
	NO	YES	1.17 (0.92, 1.49)	1.22 (0.96, 1.57)	1.21 (0.95, 1.55)	1.24 (0.97, 1.59)
	YES	NO	0.95 (0.73, 1.23)	0.99 (0.75, 1.29)	0.97 (0.74, 1.27)	0.99 (0.76, 1.30)
	YES	YES	0.60 (0.45, 0.80)***	0.64 (0.47, 0.85)**	0.62 (0.47, 0.83)**	0.64 (0.48, 0.86)**
Female						
	Iron	*Calcium*				
	NO	NO	1.00	1.00	1.00	1.00
	NO	YES	0.71 (0.48, 1.04)	0.74 (0.48, 1.15)	0.70 (0.47, 1.04)	0.74 (0.48, 1.14)
	YES	NO	1.19 (0.38, 3.75)	1.24 (0.39, 3.96)	1.18 (0.37, 3.73)	1.23 (0.39, 3.94)
	YES	YES	0.64 (0.42, 0.98)*	0.68 (0.43, 1.08)	0.63 (0.41, 0.97)*	0.67 (0.42, 1.07)
	Iron	*Folic acid*				
	NO	NO	1.00	1.00	1.00	1.00
	NO	YES	0.69 (0.44, 1.08)	0.82 (0.49, 1.35)	0.69 (0.44, 1.08)	0.81 (0.49, 1.34)
	YES	NO	0.27 (0.03, 2.10)	0.31 (0.04, 2.49)	0.26 (0.03, 2.09)	0.31 (0.04, 2.47)
	YES	YES	0.64 (0.40, 1.03)	0.78 (0.45, 1.35)	0.63 (0.39, 1.04)	0.77 (0.44, 1.34)
	Iron	*Multivitamin*				
	NO	NO	1.00	1.00	1.00	1.00
	NO	YES	0.92 (0.64, 1.33)	0.99 (0.68, 1.44)	0.96 (0.66, 1.40)	1.00 (0.68, 1.46)
	YES	NO	0.80 (0.55, 1.17)	0.87 (0.59, 1.28)	0.83 (0.57, 1.22)	0.87 (0.59, 1.29)
	YES	YES	0.89 (0.62, 1.28)	0.97 (0.67, 1.41)	0.93 (0.64, 1.34)	0.98 (0.67, 1.43)

[a]: model 1 adjusted for child's age, mode of delivery, parents' age at the childbirth, maternal prepregnancy BMI, marital status, parents' education level, family income, whether the only child, gestational diabetes mellitus, patterns and duration of breastfeeding, frequency of outdoor activities at age 0-1 years, outdoor activities of time 0-1 years of age, nutritional status of 0-1 years old, frequency of outdoor activities at age 1-3 years, outdoor activities of time 1-3 years of age and nutritional status of 1-3 years old in models.

[b]: model 2 adjusted for variables in model 1 and one other nutrient in addition to these two. The specific types of nutrients adjusted from top to bottom were: combined effect of iron and calcium adjusted folic acid; combined effects of iron and folic acid adjusted calcium; combined effects of calcium and folic acid adjusted iron; combined effect of iron and multivitamin adjusted calcium; combined effect of calcium and multivitamin adjusted iron;

combined effect of folic acid and multivitamin adjusted iron;

[c]: model 3 adjusted for variables in model 1 and the other nutrient that was not adjusted in model 2. The specific types of nutrients adjusted from top to bottom were: combined effect of iron and calcium adjusted multivitamin; combined effects of iron and folic acid adjusted multivitamin; combined effects of calcium and folic acid adjusted multivitamin; combined effect of iron and multivitamin adjusted folic acid; combined effect of calcium and multivitamin adjusted folic acid; combined effect of folic acid and multivitamin adjusted calcium;

[d]: model 4 adjusted for variables in model 1 and the other two nutrients.

Table S9: Coefficient of contingency between different nutrients.

**Coefficient of contingency**	Iron	Calcium	Folic acid	Multivitamin
Iron	/	0.226	0.168	0.239
Calcium	0.226	/	0.384	0.223
Folic acid	0.168	0.384	/	0.205
Multivitamin	0.239	0.223	0.205	/

Table S10: Sensitivity analysis of association between maternal nutrients supplementation and obesity in preschoolers born macrosomia including missing data.

Nutrients Supplementation	Total, N = 6936	Obesity (N, %)	OR (95% CI)	AOR (95% CI) [a]	AOR (95% CI) [b]
Iron					
No	4403	687 (15.60)	1.00	1.00	1.00
Yes	2533	301 (11.88)	0.73 (0.63, 0.84)***	0.77 (0.65, 0.90)***	0.80 (0.68, 0.94)**
Calcium					
No	1056	198 (18.75)	1.00	1.00	1.00
Yes	5880	790 (13.44)	0.67 (0.57, 0.80)***	0.72 (0.59, 0.87)***	0.79 (0.64, 0.98)*
Folic acid					
No	693	135 (19.48)	1.00	1.00	1.00
Yes	6243	853 (13.66)	0.65 (0.54, 0.80)***	0.75 (0.59, 0.94)*	0.88 (0.68, 1.14)
Multivitamin					
No	4249	638 (15.02)	1.00	1.00	1.00
Yes	2687	350 (13.03)	0.85 (0.74, 0.97)*	0.93 (0.80, 1.09)	1.03 (0.88, 1.22)

[a]: adjusted for child's sex, child's age, mode of delivery, parents' age at the childbirth, maternal prepregnancy BMI, marital status, parents' education level, family income, whether the only child, gestational diabetes mellitus, patterns and duration of breastfeeding, frequency of outdoor activities at age 0-1 years, outdoor activities of time 0-1 years of age, nutritional status of 0-1 years old, frequency of outdoor activities at age 1-3 years, outdoor activities of time 1-3 years of age and nutritional status of 1-3 years old in models.

[b]: adjusted for child's sex, child's age, mode of delivery, parents' age at the childbirth, maternal prepregnancy BMI, marital status, parents' education level, family income, whether the only child, gestational diabetes mellitus, patterns and duration of breastfeeding, frequency of outdoor activities at age 0-1 years, outdoor activities of time 0-1 years of age, nutritional status of 0-1 years old, frequency of outdoor activities at age 1-3 years, outdoor activities of time 1-3 years of age, nutritional status of 1-3 years old, and other nutrients in models.

$^{*}p < 0.05$, $^{**}p < 0.01$, $^{***}p < 0.001$.

Table S11: Sensitivity analysis of combined effects of maternal nutrients supplementation on obesity in preschoolers born macrosomia including missing data.

Nutrients Supplementation		*AOR* (95% *CI*) [a]	*AOR* (95% *CI*) [b]	*IOR* (95% *CI*) [b]	*RERI* (95% *CI*) [b]	*AP* (95% *CI*) [b]
Iron	*Calcium*			0.57 (0.31, 1.02)	-0.55 (-1.35, 0.24)	-0.84 (-2.02, 0.33)
NO	NO	1.00	1.00			
NO	YES	0.81 (0.66, 0.99)*	0.85 (0.67, 1.07)			
YES	NO	1.29 (0.74, 2.27)	1.36 (0.77, 2.39)			
YES	YES	0.62 (0.50, 0.78)***	0.65 (0.51, 0.85)**			
Iron	*Folic acid*			1.20 (0.51, 2.81)	0.16 (-0.42, 0.75)	0.23 (-0.63, 1.09)
NO	NO	1.00	1.00			
NO	YES	0.77 (0.60, 0.98)*	0.87 (0.66, 1.14)			
YES	NO	0.60 (0.26, 1.36)	0.67 (0.29, 1.55)			
YES	YES	0.61 (0.47, 0.80)***	0.70 (0.51, 0.95)*			
Iron	*Multivitamin*			0.72 (0.52, 0.99)*	-0.32 (-0.65, 0.01)	-0.41 (-0.84, 0.02)
NO	NO	1.00	1.00			
NO	YES	1.09 (0.89, 1.32)	1.17 (0.96, 1.44)			
YES	NO	0.86 (0.70, 1.07)	0.93 (0.75, 1.16)			
YES	YES	0.72 (0.58, 0.89)**	0.79 (0.63, 0.98)*			

[a]: adjusted for child's sex, child's age, mode of delivery, parents' age at the childbirth, maternal prepregnancy BMI, marital status, parents' education level, family income, whether the only child, gestational diabetes mellitus, patterns and duration of breastfeeding, frequency of outdoor activities at age 0-1 years, outdoor activities of time 0-1 years of age, nutritional status of 0-1 years old, frequency of outdoor activities at age 1-3 years, outdoor activities of time 1-3 years of age and nutritional status of 1-3 years old in models.

[b]: adjusted for child's sex, child's age, mode of delivery, parents' age at the childbirth, maternal prepregnancy BMI, marital status, parents' education level, family income, whether the only child, gestational diabetes mellitus, patterns and duration of breastfeeding, frequency of outdoor activities at age 0-1 years, outdoor activities of time 0-1 years of age, nutritional status of 0-1 years old, frequency of outdoor activities at age 1-3 years, outdoor activities of time 1-3 years of age, nutritional status of 1-3 years old, and other nutrients in models.

* $p < 0.05$, ** $p < 0.01$, *** $p < 0.001$.

Table S12: Sensitivity analysis of association between maternal nutrients supplementation and obesity in preschoolers born macrosomia after stratification by sex including missing data.

**Sex**	**Nutrients Supplementation**	**Total**	**Obesity (N, %)**	**OR (95% CI)**	**AOR (95% CI)** [a]	**AOR (95% CI)** [b]
Male						
	Iron					
	No	2784	469 (16.85)	1.00	1.00	1.00
	Yes	1491	185 (12.41)	0.70 (0.58, 0.84)***	0.72 (0.59, 0.87)***	0.74 (0.61, 0.91)**
	Calcium					
	No	675	128 (18.96)	1.00	1.00	1.00
	Yes	3600	526 (14.61)	0.73 (0.59, 0.91)**	0.75 (0.59, 0.95)*	0.85 (0.65, 1.10)
	Folic acid					
	No	421	84 (19.95)	1.00	1.00	1.00
	Yes	3854	570 (14.79)	0.70 (0.54, 0.90)**	0.74 (0.55, 0.99)*	0.85 (0.62, 1.18)
	Multivitamin					
	No	2639	427 (16.18)	1.00	1.00	1.00
	Yes	1636	227 (13.88)	0.83 (0.70, 0.99)*	0.92 (0.76, 1.12)	1.03 (0.84, 1.26)
Female						
	Iron					
	No	1619	218 (13.47)	1.00	1.00	1.00
	Yes	1042	116 (11.13)	0.81 (0.63,1.02)	0.89 (0.68, 1.17)	0.95 (0.72, 1.26)
	Calcium					
	No	381	70 (18.37)	1.00	1.00	1.00
	Yes	2280	264 (11.58)	0.58 (0.44, 0.78)***	0.66 (0.47, 0.92)*	0.69 (0.46, 1.01)
	Folic acid					
	No	272	51 (18.75)	1.00	1.00	1.00
	Yes	2389	283 (11.85)	0.58 (0.42, 0.82)**	0.74 (0.49, 1.10)	0.90 (0.57, 1.42)
	Multivitamin					
	No	1610	211 (13.11)	1.00	1.00	1.00
	Yes	1051	123 (11.70)	0.88 (0.69, 1.11)	0.98 (0.74, 1.28)	1.06 (0.80, 1.41)

[a]: adjusted for child's age, mode of delivery, parents' age at the childbirth, maternal prepregnancy BMI, marital status, parents' education level, family income, whether the only child, gestational diabetes mellitus, patterns and duration of breastfeeding, frequency of outdoor activities at age 0-1 years, outdoor activities of time 0-1 years of age, nutritional status of 0-1 years old, frequency of outdoor activities at age 1-3 years, outdoor activities of time 1-3 years of age and nutritional status of 1-3 years old in models.

[b]: adjusted for child's age, mode of delivery, parents' age at the childbirth, maternal prepregnancy BMI, marital status, parents' education level, family income, whether the only child, gestational diabetes mellitus, patterns and duration of breastfeeding, frequency of outdoor activities at age 0-1 years, outdoor activities of time 0-1 years of age, nutritional status of 0-1 years old, frequency of outdoor activities at age 1-3 years, outdoor activities of time 1-3 years of age, nutritional status of 1-3 years old, and other nutrients in models.

$^{*}p < 0.05$, $^{**}p < 0.01$, $^{***}p < 0.001$.

Table S13: Sensitivity analysis of combined effects of maternal nutrients supplementation on obesity in preschoolers born macrosomia after stratification by sex including missing data.

Sex	Nutrients Supplementation		AOR (95% CI) [a]	AOR (95% CI) [b]	IOR (95% CI) [b]	RERI (95% CI) [b]	AP (95% CI) [b]
Male							
	Iron	*Calcium*			0.48 (0.23, 0.97)*	-0.75 (-1.79, 0.29)	-1.15 (-2.68, 0.38)
	NO	NO	1.00	1.00			
	NO	YES	0.87 (0.68, 1.12)	0.93 (0.70, 1.23)			
	YES	NO	1.39 (0.71, 2.72)	1.48 (0.75, 2.92)			
	YES	YES	0.62 (0.46, 0.82)***	0.65 (0.48, 0.90)**			
	Iron	*Folic acid*			0.78 (0.30, 2.05)	-0.17 (-1.10, 0.75)	-0.27 (-1.67, 1.14)
	NO	NO	1.00	1.00			
	NO	YES	0.80 (0.59, 1.09)	0.88 (0.62, 1.23)			
	YES	NO	0.86 (0.34, 2.19)	0.94 (0.37, 2.43)			
	YES	YES	0.58 (0.42, 0.82)**	0.65 (0.44, 0.95)*			
	Iron	*Multivitamin*			0.54 (0.36, 0.81)**	-0.58 (-1.01, -0.15)	-0.85 (-1.54, -0.17)**
	NO	NO	1.00	1.00			
	NO	YES	1.19 (0.94, 1.51)	1.28 (1.00, 1.63)			
	YES	NO	0.92 (0.71, 1.19)	0.98 (0.75, 1.28)			
	YES	YES	0.63 (0.47, 0.83)**	0.68 (0.51, 0.91)**			
Female							
	Iron	*Calcium*			0.76 (0.25, 2.30)	-0.28 (-1.61, 1.06)	-0.42 (-2.39, 1.56)
	NO	NO	1.00	1.00			
	NO	YES	0.68 (0.47, 0.99)*	0.71 (0.47, 1.08)			
	YES	NO	1.19 (0.41, 3.44)	1.23 (0.42, 3.60)			
	YES	YES	0.64 (0.43, 0.96)*	0.66 (0.42, 1.04)			
	Iron	*Folic acid*			3.74 (0.46, 30.34)	0.72 (0.13, 1.31)**	0.90 (0.02, 1.78)*
	NO	NO	1.00	1.00			
	NO	YES	0.68 (0.45, 1.04)	0.81 (0.5, 1.31)			
	YES	NO	0.23 (0.03, 1.77)	0.26 (0.03, 2.11)			
	YES	YES	0.65 (0.42, 1.02)	0.80 (0.47, 1.36)			
	Iron	*Multivitamin*			1.16 (0.67, 2.02)	0.15 (-0.38, 0.67)	0.14 (-0.36, 0.64)
	NO	NO	1.00	1.00			
	NO	YES	0.90 (0.63, 1.30)	0.99 (0.68, 1.44)			
	YES	NO	0.80 (0.56, 1.16)	0.89 (0.61, 1.30)			
	YES	YES	0.92 (0.65, 1.30)	1.03 (0.71, 1.48)			

[a]: adjusted for child's age, mode of delivery, parents' age at the childbirth, maternal prepregnancy BMI, marital status, parents' education level, family income, whether the only child, gestational diabetes mellitus, patterns and duration of breastfeeding, frequency of outdoor activities at age 0-1 years, outdoor activities of time 0-1 years of age, nutritional status of 0-1 years old, frequency of outdoor activities at age 1-3 years, outdoor activities of time 1-3 years of age and nutritional status of 1-3 years old in models.

[b]: adjusted for child's age, mode of delivery, parents' age at the childbirth, maternal prepregnancy BMI, marital status, parents' education level, family income, whether the only child, gestational diabetes mellitus, patterns and duration of breastfeeding, frequency of outdoor activities at age 0-1 years, outdoor activities of time 0-1 years of

age, nutritional status of 0-1 years old, frequency of outdoor activities at age 1-3 years, outdoor activities of time 1-3 years of age, nutritional status of 1-3 years old, and other nutrients in models.

* $p < 0.05$, ** $p < 0.01$, *** $p < 0.001$.
